# Visible emission from Ce-doped ZnO nanorods grown by hydrothermal method without a post thermal annealing process

**DOI:** 10.1186/1556-276X-7-43

**Published:** 2012-01-05

**Authors:** Yong-Il Jung, Bum-Young Noh, Young-Seok Lee, Seong-Ho Baek, Jae Hyun Kim, Il-Kyu Park

**Affiliations:** 1 LED-IT Fusion Technology Research Center and Department of Electronic Engineering, Yeungnam University, Gyeongbuk, 712-749, Korea; 2 Energy Research Division, Daegu Gyeongbuk Institute of Science & Technology(DGIST), 50-1, Sang-Ri, Hyeonpung-Myeon, Dalseong-gun, Daegu, 711-873, Korea

**Keywords:** nanostructures, oxides, crystal growth, optical properties

## Abstract

Visible light-emitting Ce-doped ZnO nanorods [NRs] without a post thermal annealing process were grown by hydrothermal method on a Si (100) substrate at a low temperature of 90°C. The structural investigations of Ce-doped ZnO NRs showed that the Ce^3+ ^ions were successfully incorporated into the ZnO lattice sites without forming unwanted Ce-related compounds or precipitates. The optical investigation by photoluminescence spectra shows that the doped Ce^3+ ^ions in the ZnO NRs act as an efficient luminescence center at 540 nm which corresponds to the optical transition of 5*d *→ 4*f *orbitals in the Ce^3+ ^ions. The photoluminescence intensity of the Ce-doped ZnO NRs increased with the increasing content of the Ce-doping agent because the energy transfer of the excited electrons in ZnO to the Ce^3+ ^ions would be enhanced by increased Ce^3+ ^ions.

## Introduction

Zinc oxide [ZnO] nanostructures have received much attention due to their remarkable performance in electronic, piezoelectric, thermoelectric, and optoelectronic applications [1]. High electrical conductivity and transparency in a visible wavelength spectral range of the ZnO alloys have been regarded as an efficient candidate for transparent conducting electrodes of thin films and flexible electronics [2]. The absence of a center of symmetry in its wurtzite structure, along with a large electromechanical coupling, results in strong piezoelectric and pyroelectric properties which make it an energy recycling material system [3]. In addition, because ZnO has a large direct bandgap of 3.37 eV and a high exciton binding energy of 60 meV, it has been much investigated for optoelectronic applications, such as light-emitting diode [LED] and UV laser diode [4]. Recently, the ZnO nanostructures doped with rare earth elements such as Ce, Y, and Eu have achieved much attention for biological tagging as well as optoelectronic applications due to their unique optical properties [5-7]. Most of all, the Ce element possessing a unique optical characteristic may be an ideal material for visible light-emitting phosphors in display, high-power laser, and light-emitting diode [5-9]. However, few have been reported for the fabrication and optical properties of the Ce-doped ZnO nanostructures especially by hydrothermal method [7-9] even though this method has many advantages of low temperature process which makes it favorable to the integration and *in-situ *fabrication of various devices. Furthermore, for more compatibility of the Ce-doped ZnO nanorods [NRs] to be applicable to other devices, visible emission should be possible without post thermal annealing processes. In this report, we have fabricated visible light-emitting Ce-doped ZnO NRs by hydrothermal method without a post thermal annealing process.

## Experimental details

### Growth of undoped and Ce-doped ZnO NRs on Si (100) substrate

The Ce-doped ZnO NRs were grown on a *p*-type Si (100) substrate. After cleaning the Si substrate by acetone, methanol, HF, and deionized [DI] water, a seed layer for the ZnO NRs was formed by dipping the substrate into 40 mM of zinc acetate dihydrate (Zn(CH_3_COO)_2_•2H_2_O) dissolved in ethanol solution, followed by drying at 100°C for 5 min. The Ce-doped ZnO NRs were grown by placing the seed layer grown substrate into a mixed solution of 20 mM zinc nitrate hexahydrate (Zn(NO_3_)_2_•6H_2_O), 20 mM hexamethylenetetramine [HMT] ((CH_2_)_6_N_4_), and cerium nitrate hexahydrate (Ce(NO_3_)_3_•6H_2_O) in DI water at 90°C for 3 h. The amount of Ce agent was varied from 0.1 to 0.8 mM, which corresponds from 0.5% to 4% in molarity. At a solution temperature above 70°C, the solution started being cloudy, indicating that a chemical reaction started. After the reaction, the samples were cleaned in the flowing DI water for 5 min.

### Structural characterizations

The structural properties of Ce-doped ZnO NRs were investigated by field emission scanning electron microscopy [FE-SEM], energy dispersive X-ray spectroscopy [EDS], and X-ray diffraction [XRD] with an excitation source of Cu Kα radiation. The chemical composition of the deposited ZnO NRs was observed using EDS attached to a FE-SEM microscope.

### Optical characterizations

The optical properties of Ce-doped ZnO NRs were investigated by photoluminescence [PL] spectra which were measured by using a 24-mW power 325-nm continuous He-Cd laser at room temperature. The laser was focused onto the sample surface by an objective lens; the excitation area was estimated to be about 400 μm in diameter.

## Results and discussion

**Figure 1:**
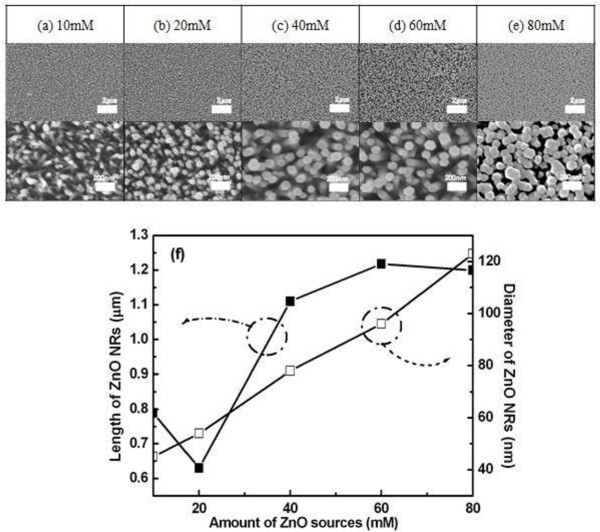
**FE-SEM images of ZnO NRs grown by various ZnO source molarities**. ZnO source molarities of (**a**) 10 mM, (**b**) 20 mM, (**c**) 40 mM, (**d**) 60 mM, (**e**) 80 mM in the main solution and (**f**) Variation of the ZnO NR size with variation of source molarities.

Figures 1a to 1e compare the FE-SEM images of undoped ZnO NRs grown on Si substrate with an increasing amount of ZnO sources. As the molar content of the ZnO source increases to 80 mM, the well-faceted six-side surfaces are developed, and the top of the ZnO NRs shows a hexagonal facet, indicating that the ZnO NRs are single crystals grown along the [0001] direction [10]. The average length and diameter of the ZnO NRs obtained from FE-SEM images were plotted versus the ZnO source molar contents in Figure 1f. With the increasing molar content of the ZnO source from 10 to 80 mM, the diameter increased linearly from 45 to 123 nm. The length of the ZnO NRs decreased, with the source molar content up to 20 mM, then increased up to 60 mM, and finally, saturated again. In this way, we could control the size and morphologies of ZnO NRs by molar contents of sources in the main solution. This result indicates that the growth mechanism of the ZnO NRs is governed by the source supplying rate from the solution to the growth front surface of ZnO NRs, not by the surface reaction's limited condition when the source molar contents were below 60 mM. Therefore, in the source molar content range below 60 mM, the Ce dopant atoms can be incorporated into the ZnO matrix in a controllable way because the growth is governed by the source supplying rate.

**Figure 2:**
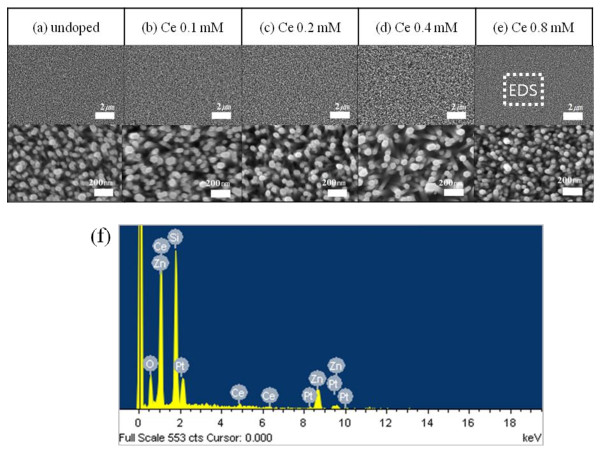
**FE-SEM images and EDS patterns of Ce-doped ZnO NRs**. FE-SEM images of (**a**) undoped, (**b**) 0.1-mM (0.5%), (**c**) 0.2-mM (1%), (**d**) 0.4-mM (2%), and (**e**) 0.8-mM (4%) Ce-doped ZnO NRs. (**f**) EDS patterns of 0.8-mM Ce-doped ZnO NRs.

Figures 2a to 2e show the surface morphologies of the Ce-doped ZnO with different amounts of the Ce agent, from 0.1 to 0.8 mM, in the ZnO NRs which were grown by 20 mM of ZnO source agent. The diameter of the undoped ZnO NRs is nearly 50 nm. As shown in Figures 2b to 2e, the morphology of ZnO NRs did not change significantly with the changing amount of the Ce dopant agent. This indicates that the growth of ZnO NRs is not influenced by the doping of Ce atoms because the Ce(NO_3_)_3_•6H_2_O agent was used for supplying Ce^3+ ^ions to be doped, and the molar contents of the dopant agent, compared to the ZnO source, was too small to change the morphology. Figure 2f is an EDS pattern of the 0.8-mM Ce-doped ZnO NRs showing that there are Zn, O, and Ce elements in the ZnO NRs. The other peaks noted Si and Pt to correspond to the substrate and coating material for SEM measurement. The EDS results show that the amount of Ce in ZnO NRs is 2.6%, indicating that the Ce dopant is successfully incorporated into the ZnO NRs. The mechanism for the synthesis of the Ce-doped ZnO NRs using HMT can be summarized in the following equations:

$$\begin{array}{c} {\left(\text{C}{\text{H}}_{\text{2}}\right)}_{\text{6}}{\text{N}}_{\text{4}}+\text{ 6}{\text{H}}_{\text{2}}\text{O}\to \text{6CO}{\text{H}}_{\text{2}}+\text{ 4N}{\text{H}}_{\text{3}}\\ \text{N}{\text{H}}_{\text{3}}+\;{\text{H}}_{\text{2}}\text{O}\to \text{N}{\text{H}}_{\text{3}}\cdot {\text{H}}_{\text{2}}\text{O}\to \text{N}{{\text{H}}_{\text{4}}}^{+}+\text{ O}{\text{H}}^{-}\\ \text{Zn}{\left(\text{N}{\text{O}}_{\text{3}}\right)}_{\text{2}}\cdot \text{6}{\text{H}}_{\text{2}}\text{O }\to \text{ Z}{\text{n}}^{\text{2}+}+\text{ 2N}{{\text{O}}_{\text{3}}}^{-}+\text{ 6}{\text{H}}_{\text{2}}\text{O}\text{.}\\ \text{Z}{\text{n}}^{\text{2}+}+\text{ 2O}{\text{H}}^{-}\to \text{ZnO }+\;{\text{H}}_{\text{2}}\text{O}\\ \text{Ce}{\left(\text{N}{\text{O}}_{\text{3}}\right)}_{\text{3}}\cdot \text{6}{\text{H}}_{\text{2}}\text{O }\to \text{ C}{\text{e}}^{\text{3}+}+\text{ 3N}{{\text{O}}_{\text{3}}}^{-}+\text{ 6}{\text{H}}_{\text{2}}\text{O}\\ \text{4C}{\text{e}}^{\text{3}+}+\text{ 12O}{\text{H}}^{-}\to \text{ 2C}{\text{e}}_{\text{2}}{\text{O}}_{\text{3}}+\text{ 6}{\text{H}}_{\text{2}}\text{O} \end{array}$$

HMT plays a very complicated role in the solution during the hydrothermal process [11], but it supplies OH^- ^ions to the Zn^2+ ^and Ce^3+ ^ions to form Zn-O and Ce-O bonds here, respectively. Thereby, Ce^3+ ^ions substitute the Zn lattice sites during the growth of ZnO NRs.

**Figure 3:**
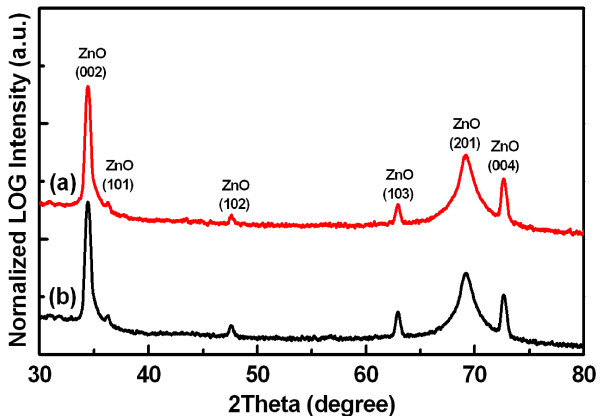
**Normalized *θ-*2*θ *XRD scan spectra**. (**a**) Undoped and (**b**) 0.8-mM Ce-doped ZnO NRs in log scale.

Figures 3a and 3b show the XRD patterns of as-prepared undoped ZnO NRs and 0.8-mM Ce-doped ZnO NRs, respectively. The diffraction peaks are quite similar to those of bulk ZnO. The diffraction peaks in the pattern can be indexed to the standard hexagonal wurtzite-structured ZnO (space group: P63mc; *a *= 0.3249 nm, *c *= 0.5206 nm), and diffraction data are in agreement with the JCPDS card for ZnO (JCPDS 36-1451). All the diffraction peaks were identified as corresponding to (002), (101), (102), (103), (201), and (004) planes of the hexagonal wurtzite ZnO structure, and no additional peaks corresponding to Ce-related alloys were observed as shown in Figure 3b. This result shows that the as-prepared products are single-phase hexagonal ZnO, and the Ce-related compounds or precipitates are not formed during growth, but the Ce atoms are incorporated into the lattice sites in the ZnO. Even though the ionic radius of Ce^3+ ^(1.03 Å) is bigger than that of Zn^2+ ^(0.74 Å), there is no change in diffraction peak positions between the undoped and the 0.8-mM Ce-doped ZnO NRs because the amount of incorporated Ce dopants is negligibly small to be detectable in the diffraction data.

**Figure 4:**
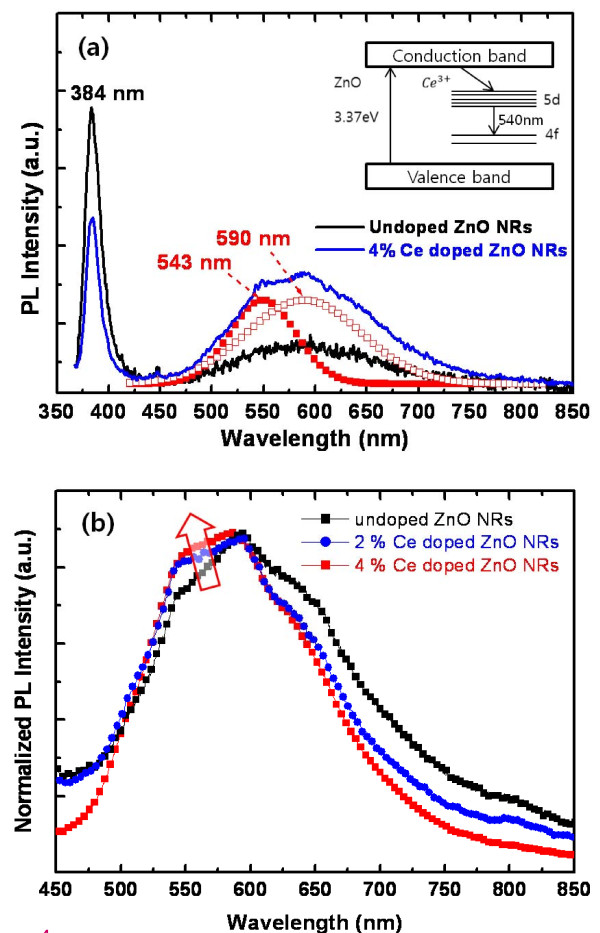
**Normalized PL sprectra measured at room temperature**. (**a**) Room temperature PL spectra of as-grown undoped and Ce-doped ZnO NRs. Two elementary PL spectra shown in dotted lines are obtained by deconvolution of the PL spectrum of doped ZnO NRs. The inset shows a schematic energy level diagram of Ce^3+ ^electronic levels in ZnO bandgap. (**b**) Normalized PL spectra of ZnO NRs doped with different amounts of Ce molar contents.

Figure 4a shows the PL spectrum of as-grown undoped ZnO NRs and 0.8-mM Ce-doped ZnO NRs measured at room temperature. The PL spectra of both samples exhibit mainly two emission peaks at UV and visible ranges, which correspond to the band edge emission of ZnO and deep or Ce-activated atomic levels, respectively. The emission band at a visible wavelength range of the undoped ZnO NRs arises from a radiative recombination through point defects in the ZnO lattice, such as oxygen vacancy, zinc vacancy, oxygen interstitial, zinc interstitial, and anti-site defects, as reported by many other papers [12,13]. The PL peak positions of the UV emission for undoped and Ce-doped ZnO NRs are the same at 384 nm. If the Ce dopant incorporated into the ZnO as a donor with the valence state Ce^4+^, the band edge emission peak position from Ce-doped ZnO NRs should be red-shifted [9]. The undoped ZnO NR shows a larger UV/visible emission intensity ratio than that of the Ce-doped ZnO NRs. Therefore, these indicate that the Ce^3+ ^ion activators are embedded in the ZnO host matrix and act as a luminescence center in the visible spectral range. The emission band at a visible range of the Ce-doped ZnO NRs is composed of two elementary PL peaks at 543 and 590 nm. The PL peak at 590 nm corresponds to the emission by the mentioned point defects, while the peak at 543 nm is from the emission by electron energy transition from 5*d *to 4*f *orbitals in Ce^3+ ^ions, as shown in the inset of Figure 4a. The fluorescence of Ce^3+^-activated compounds usually consists of a broad band with two peaks because the ground state of the Ce^3+ ^ion consists of a doublet (^2^F_5/2 _and ^2^F_7/2_) [14]. To investigate the effect of the Ce dopant amount on the optical properties, PL spectra were measured precisely in the visible range by enhancing the sensitivity of the detector as shown in Figure 4b. The PL intensity at 543 nm increases gradually with the increase of the molar ratio of the Ce dopant agent. The energy of excited electrons in the conduction band of the ZnO is transferred to the Ce^3+ ^ion activators to luminescence. Therefore, with the increase of Ce^3+ ^ions in the ZnO matrix, the energy transfer rate of excited electrons to Ce^3+ ^ions can be enhanced, resulting in the increase of the PL intensity at this wavelength. A visible emission from the ZnO NRs without a post thermal annealing process is a very important point here because it makes possible for the ZnO NRs to be applicable with the active layer or wavelength conversion layers of optoelectronic devices, such as visible wavelengths or white LEDs.

## Conclusions

In summary, Ce-doped ZnO NRs were grown on Si substrates by using the hydrothermal method. The structural properties investigated by FE-SEM, EDS, and XRD showed that the Ce^3+ ^ions are successfully incorporated into the ZnO lattice sites; the growth of ZnO NRs was not influenced by the doping of Ce atoms because the molar content of the dopant was too small an amount to change the morphology, and the dopant agent provided Ce^3+ ^ions for doping. The XRD results showed that the ZnO NRs are single-phase hexagonal ZnO, and unwanted Ce-related compounds or precipitates were not formed during the growth of Ce-doped ZnO NRs. The PL results showed that the doped Ce^3+ ^ions in the ZnO NRs act as a luminescence center for visible emission at 543 nm even though the ZnO NRs were not thermally annealed. The PL intensity at a visible range of the Ce-doped ZnO NRs increased with the increased molarity of the Ce dopant agent because the energy transfer rate of the excited electrons from the conduction band in the ZnO host to a 5*d *energy state in the Ce^3+ ^ion activators could be enhanced by increased Ce^3+ ^ions. These results demonstrate that Ce doping in ZnO NRs can be an efficient luminescence center in ZnO NRs without post thermal annealing processes.

## Competing interests

The authors declare that they have no competing interests.

## Authors' contributions

YIJ and IKP developed the concept and design of the Ce-doped ZnO nanorods. YIJ carried out the fabrication of the Ce-doped ZnO nanorods. BYN carried out the structural characterization of the ZnO nanorods. YSL, SHB, and JHK carried out the optical characterization by photoluminescence measurement. YIJ and IKP analyzed the results and wrote the manuscript. All authors read and approved the final manuscript.
